# Novel Treatment for a Giant Acrochordon Using Pachaieruvai, a Traditional Siddha Medicine: A Case Report

**DOI:** 10.7759/cureus.82922

**Published:** 2025-04-24

**Authors:** Saravanasingh Karan Chand Mohan Singh, A Jayakalaiarasi, Shalini Boopathi, V Gowri, Sathiya Vaidhyanathan

**Affiliations:** 1 Department of Maruthuvam (Integrative/Complementary Medicine), National Institute of Siddha, Chennai, IND; 2 Department of Sattam Sarntha Maruthuvamum Nanju Noolum (Forensic Medicine and Toxicology), Santhigiri Siddha Medical College, Trivandrum, IND; 3 Department of Kuzhanthai Maruthuvam (Pediatrics), National Institute of Siddha, Chennai, IND; 4 Department of Gunapadam-Marunthakaviyal (Pharmaceuticals), Maria Siddha Medical College and Hospital, Tamil Nadu, IND; 5 Department of Udal Koorugal (Anatomy), JSA Medical College for Siddha and Research Centre, Ulundurpet, IND

**Keywords:** case report, giant acrochordons, pachaieruvai, siddha medicine, skin tags

## Abstract

Skin tags, also known as acrochordons, are common benign growths that often appear in areas where the skin experiences friction. One of the challenges for healthcare providers is finding more targeted treatment options for skin tags, as current methods-like electrodesiccation, laser therapy, cryotherapy, and surgical removal-can be quite expensive. Fortunately, Siddha medicine offers a promising and potentially more affordable alternative. In this case report, we discuss the successful treatment of a giant acrochordon in a 47-year-old male patient using Pachaieruvai Siddha medicine, an indigenous healing tradition from India. The patient applied Pachaieruvai externally for 10 days and experienced only mild, temporary discomfort. Substantial lesion disappearance was observed within two weeks, and subsequent evaluations at three and six months showed no recurrence. These results suggest that Siddha pharmacotherapy may serve as an effective, less invasive, and economical alternative for the management of acrochordons. However, further research and clinical studies are essential to validate its efficacy and develop standardized treatment protocols.

## Introduction

Acrochordons, commonly known as skin tags, are pedunculated, soft, movable, often flesh-colored, and primarily appear on skin in areas where there’s friction or where the skin folds, like the neck, armpits, and groin [1]. While these growths are generally harmless, they can lead to cosmetic concerns and, in some cases, physical discomfort or irritation [2]. Acrochordons are particularly prevalent among middle-aged and older individuals, and they tend to be associated with conditions such as obesity, diabetes, hyperlipidemia, insulin resistance, acromegaly, mechanical friction, and metabolic syndrome [3,4]. There is a strong link between acrochordons and obesity. In fact, studies show that 74% of patients with these skin tags are either obese or overweight, and about 24% meet the criteria for metabolic syndrome [5].

Human papillomavirus (HPV) has been found in around 80% of skin tags, with subtypes 6 and 11 being the most common. This finding suggests that HPV might play a role in the development of these skin growths, although the precise mechanism behind this connection is still not fully understood [6,7]. Acrochordons are typically diagnosed through clinical examination, and physical assessment is sufficient for identification [8]. When it comes to treatment options, conventional methods often include surgical excision or cryotherapy. However, it's important to note that these approaches can carry risks, such as scarring, infection, and recurrence of the tags [9].

This case report delves into the promising potential of Siddha medicine, an indigenous Indian healing tradition, focusing particularly on Pachaieruvai as a viable and cost-effective alternative for managing acrochordon. Siddha pharmacotherapy is a unique aspect of the Siddha medical system, known for its use of specially processed metals and minerals such as mercury, silver, arsenic, lead, and sulfur in treating a variety of ailments. These formulations have a long-standing history of being used to address infectious conditions while minimizing adverse effects. Moreover, Siddha medicine has also shown effectiveness in treating cancer, psoriasis, chronic liver diseases, infertility, benign prostatic hyperplasia, bleeding hemorrhoids, peptic ulcers, and various skin conditions [3].

## Case presentation

A 47-year-old man presented to our outpatient clinic with a single, sizable, soft, skin-colored growth located on the back over the right hip joint. The lesion has been present for the past 10 years, gradually increasing in size. The patient reported no associated symptoms such as pain, itching, or bleeding, but noted occasional friction with clothing due to the growth's size. He had no significant medical history, including hypertension, diabetes, or other systemic illnesses. On examination, the lesion was identified as a large pedunculated skin tag (acrochordon) measuring approximately 10 cm in diameter. It was soft, non-tender, and freely movable, attached to the skin by a narrow stalk. The overlying skin was intact, with no signs of inflammation, ulceration, or secondary infection. The surrounding skin appeared normal, and no other similar lesions were noted elsewhere on the body.

Patient history

The patient stated that the lesion had been present for a decade, initially small and gradually enlarging over time. He denied any trauma, discharge, or changes in color associated with the growth. Despite its size, the skin tag did not cause functional limitations or discomfort beyond friction with clothing.

Diagnosis

The lesion was clinically diagnosed as a skin tag (acrochordon) based on its presentation as a large, soft, pedunculated, skin-colored growth. General and systemic examinations were unremarkable, and no other abnormalities were identified.

Management

**Figure 1:**
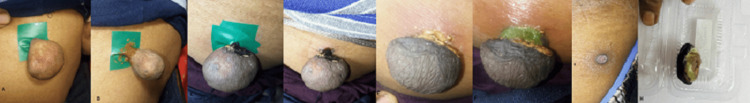
A. Giant skin tag before treatment; B. Giant skin tag on Day 1 of treatment; C. Giant skin tag on Day 3 of treatment; D. Giant skin on Day 5 of treatment; E. Giant skin on Day 7 of treatment; F. Giant skin tag on Day 10 of treatment; G. Fully destroyed giant skin tag after treatment (Day 15); H. Separated skin tag

The patient initially opted for treatment due to persistent irritation caused by friction with clothing. Instead of surgical excision, the patient chose a traditional Siddha medicine approach using Pachaieruvai, a topical herbal preparation. The medicine was administered with the prerequisite of written consent. The treatment involved daily application of the medicine over the stalk for ten consecutive days. During treatment, the patient experienced mild pain, burning sensations at the application site, which lasted for about 30 min after each application. These symptoms were temporary and resolved immediately. These reactions were interpreted as positive indicators of the lesion's regression. The treatment successfully destroyed the growth within 15 days. Figure 1 shows the progression of the skin tag.

Follow up

The patient was reassessed two weeks after completing treatment. The lesion site had healed well, with no signs of infection or residual growth. At the three-month follow-up, there was no evidence of recurrence or adverse effects, confirming the efficacy and safety of the chosen treatment regimen with Pachaieruvai. The patient remained symptom-free, and no complications were reported, highlighting the success of this alternative approach for managing a large acrochordon.

## Discussion

Acrochordons, commonly referred to as skin tags, are benign fibroepithelial growths often associated with friction-prone areas such as the neck, axilla, and groin [1]. While most skin tags are small (typically less than 5 mm), giant skin tags measuring 10 centimeters in diameter are rare. Risk factors for acrochordons include obesity, insulin resistance, and hormonal changes; however, this patient lacked any known contributing systemic conditions [4]. The significant size of the lesion in this case may be attributed to chronic mechanical stimulation. Although large acrochordons are benign, they can cause discomfort due to friction, aesthetic concerns, or secondary complications such as irritation or infection [2]. 

This case highlights the successful use of Pachaieruvai, a traditional Siddha medicinal preparation, as an effective alternative therapy for treating a large acrochordon. Rooted in ancient Siddha texts, Pachaieruvai is composed of five key ingredients: Vellaipadanam (arsenic trioxide), Aridharam (arsenic trisulfide), Thurusu (copper sulfate), Karchunnam (calcium carbonate), and Kungiliyam (resin from Shorea robusta), each with distinct pharmacological properties [10]. Research has indicated that Vellaipaadanam, commonly recognized as arsenic trioxide and an essential element of Pachaieruvai, exhibits remarkable anti-glioma and antiviral effects. Evidence suggests that it curtails the growth of glioma cells while enhancing programmed cell death [11]. Vellaipadanam and Aridharam exhibit potent cytotoxic effects, inducing apoptosis and inhibiting tumor growth [12]. Thurusu, also known as copper sulfate, exhibits notable cytotoxic properties that inhibit tumor growth and demonstrate antiviral effects. Its mechanism of action involves generating reactive oxygen species (ROS), which induce cytotoxic reactions that disrupt cellular integrity and hinder the proliferation of abnormal cells [13-15]. Karchunnam further contributes by suppressing abnormal cell growth [16]. Kungiliyam, a resin extracted from Shorea robusta, exhibits strong cytotoxic properties that inhibit abnormal cellular growth. Research has demonstrated that extracts from Shorea robusta can suppress the proliferation of cancer cells. Notably, robustic acid derivatives found in the resin have shown potent cytotoxic effects against various cancer cell lines, including HL-60 (human leukemia) and HepG2 (human hepatoblastoma) [17,18].

Mode of action

When combined, these five ingredients work together in a powerful way to specifically target the abnormal cells linked to acrochordons. The cytotoxic effects of Vellaipadanam, Aridharam, Thurusu, and Kungiliyam collaborate to promote cell death and prevent the excessive growth of these abnormal cells. Meanwhile, calcium carbonate plays a crucial role by creating an optimal environment that enhances the effectiveness of these active ingredients. Together, this unique blend not only helps reduce but can also lead to the complete elimination of skin tags by directly attacking the cells responsible for their growth, resulting in noticeable shrinkage or even disappearance of the lesion [12,13,16,17].

Limitations of the study

This single case report presents several limitations that affect the generalizability and reliability of the findings regarding Pachaieruvai for treating acrochordons. The lack of a larger sample size restricts the ability to draw robust conclusions about its effectiveness and safety across different individuals. Additionally, the absence of controlled trials makes it difficult to ascertain whether the observed results are genuinely due to the Siddha medicine or influenced by natural regression or other factors. While the patient showed no recurrence at three and six months, a longer follow-up period is essential to confirm the treatment's long-term efficacy and safety. The subjective reports of mild discomfort also highlight the variability in patient experiences, as there was no standardized measurement for these outcomes. Furthermore, potential biases in reporting outcomes and the lack of objective data, such as histological analyses, weaken the findings. The mechanisms by which Pachaieruvai's ingredients act on acrochordons remain unclear, necessitating further biochemical research. Moreover, the study points out the need for standardization in Siddha medicine, as preparation and dosage can vary significantly among practitioners. Lastly, although no immediate adverse effects were noted, the inclusion of arsenic-based compounds raises safety concerns that warrant further investigation, especially regarding the long-term risks associated with arsenic exposure.

## Conclusions

This case report shares an encouraging success story about treating a giant acrochordon using Pachaieruvai, a traditional Siddha medicine. Within just two weeks of starting the treatment, the patient saw complete resolution of a 10 cm skin tag, and follow-up appointments at three and six months confirmed there was no recurrence. What’s more, the treatment was well received, with only mild and temporary discomfort during application. This case highlights several benefits of using Siddha pharmacotherapy for acrochordons, such as its non-invasive approach, cost-effectiveness compared to surgical options, and impressive cosmetic results. However, it’s important to note that this is just one case, and we can’t draw definitive treatment guidelines from it. Moving forward, future research should include controlled clinical trials with larger groups of patients, standardizing how Pachaieruvai is prepared and applied, comparing it to conventional treatments, and assessing the safety of the arsenic compounds involved. While traditional medicines like Pachaieruvai show great promise, their use in modern medicine needs thorough scientific validation, adding to the growing evidence of Siddha medicine's valuable role in treating skin conditions.

## References

[1] Baliga MS, Dsouza JJ (2011). Amla (Emblica officinalis Gaertn), a wonder berry in the treatment and prevention of cancer. Eur J Cancer Prev.

[2] Freedman BM (2009). Topical antioxidant application augments the effects of intense pulsed light therapy. J Cosmet Dermatol.

[3] Karan Chand Mohan Singh S, Murugan R, I N, S ES, Gowri V (2024). Evaluating the efficacy of Siddha pharmacotherapy for acrochordon: a case report. Cureus.

[4] El Safoury OS, Fawzy MM, Hay RM, Hassan AS, El Maadawi ZM, Rashed LA (2011). The possible role of trauma in skin tags through the release of mast cell mediators. Indian J Dermatol.

[5] Ramya N, Prakash B, Kumar S (2015). Skin tags and its association with systemic illnesses. Journal of Evolution of Medical and Dental Sciences.

[6] Ramachandrareddy D, Prathap R (2013). Human papilloma virus (HPV) causing skin tags. Journal of Evolution of Medical and Dental Sciences.

[7] Owczarczyk-Saczonek AW, Placek W (2016). Skin tags as a symptom of metabolic disorders [article in Polish]. Przegl Dermatol.

[8] Akan S, Arslan U, Kilic EY (2024). Penile acrochordon: an unusual site of presentation and the role of the androgen receptors [IN PRINT]. Urol Case Rep.

[9] Savant SS Jr, Savant SS, Daruwala F (2024). Selective therapy (cryo or scalpel) combined with multimodal therapy for treating keloids. J Cutan Aesthet Surg.

[10] Uthamarayan KS (2013). Siddhar aruvai maruthuvam [book in Tamil]. https://archive.org/details/dli.jZY9lup2kZl6TuXGlZQdjZp1kuty.TVA_BOK_0007005/page/49/mode/1up?q=69&view=theater.

[11] Bell JB, Eckerdt F, Dhruv HD (2018). Differential response of glioma stem cells to arsenic trioxide therapy is regulated by MNK1 and mRNA translation. Mol Cancer Res.

[12] Zheng J, Deng YP, Lin C, Fu M, Xiao PG, Wu M (1999). Arsenic trioxide induces apoptosis of HPV16 DNA-immortalized human cervical epithelial cells and selectively inhibits viral gene expression. Int J Cancer.

[13] Syrkin AB, Chlenova EL (1988). [Antidotal and antineoplastic properties of copper sulfate]. Biull Eksp Biol Med.

[14] Styczynski AR, Anwar KN, Sultana H (2015). In vitro antiretroviral activity and in vivo toxicity of the potential topical microbicide copper phthalocyanine sulfate. Virol J.

[15] Chen SY, Liu ST, Lin WR, Lin CK, Huang SM (2019). The mechanisms underlying the cytotoxic effects of copper via differentiated embryonic chondrocyte gene 1. Int J Mol Sci.

[16] Maleki Dizaj S, Barzegar-Jalali M, Zarrintan MH, Adibkia K, Lotfipour F (2015). Calcium carbonate nanoparticles as cancer drug delivery system. Expert Opin Drug Deliv.

[17] Prakash E, Rao J (1999). A new flavone glycoside from the seeds of Shorea robusta. Fitoterapia.

[18] Chen R, Huo L, Jaiswal Y (2019). Synthesis and evaluation of anticancer activity of new 4-acyloxy derivatives of robustic acid. Int J Mol Sci.

